# Simulation‐free spine palliative radiotherapy enabled by AI‐adapted diagnostic CT

**DOI:** 10.1002/mp.70366

**Published:** 2026-02-26

**Authors:** Yiding Han, Alexander Nicola Hanania, Zaid Ali Siddiqui, Vincent Ugarte, Boran Zhou, Abdallah S. R. Mohamed, Piyush Pathak, Daniel Allen Hamstra, Baozhou Sun

**Affiliations:** ^1^ Radiation Oncology Department Baylor College of Medicine TX USA

## Abstract

**Background:**

Radiotherapy planning traditionally requires a dedicated simulation CT (sCT), which can introduce delays in initiating treatment. This is particularly impactful in spinal palliative care, where timely treatment is often important for symptom control and prevention of neurological deterioration. Although diagnostic CT (dCT) is frequently available earlier in the workflow, it can lead to geometric and dosimetric inaccuracies when used directly for treatment planning due to discrepancies in patient positioning, vertebral alignment, and table curvature.

**Purpose:**

To develop and evaluate an AI‐based method that transforms dCT into a simulation‐equivalent planning CT (AI‐pCT), enabling a clinically feasible simulation‐free workflow for spinal palliative radiotherapy.

**Methods:**

Two neural networks were trained to correct spine position and body contour using paired dCT–sCT images from 50 patients (42 train/validation, 8 internal tests) in a safety net hospital and externally evaluated on 7 additional academic medical center (AMC) patients. After rigid bone‐based alignment to sCT, dosimetric accuracy was assessed by comparing DVH endpoints (Dmean, Dmax, D95, D99, V100, V107) and DVH Root‐Mean‐Square (RMS) error for plans recalculated on dCT versus AI‐pCT versus sCT. Four radiation oncologists scored image suitability. Significance was evaluated using the Wilcoxon signed‐rank test.

**Results:**

In the safety net cohort, AI‐pCT substantially reduced geometric and dosimetric error relative to dCT (e.g., Dmean error 2.0%→0.57%; RMS DVH error 6.4%→2.2%, all *p* < 0.05), improved physician plan‐quality ratings from “Acceptable” to “Good–Perfect,” and increased plan‐level clinical goal achievement from 37.5% to 100%. In the AMC cohort, where baseline dCT was already closely aligned to sCT, AI‐pCT produced smaller but still statistically significant gains.

**Conclusion:**

AI‐pCT achieves sCT‐level geometric and dosimetric fidelity without requiring a separate simulation scan, enabling a simulation‐free planning workflow for spinal palliative RT. This approach has the potential to reduce treatment delays and improve access, particularly in resource‐constrained environments.

## 1 INTRODUCTION

Over half of cancer patients require radiotherapy (RT) during their disease course, and this demand continues to grow due to an aging population and expanding indications for RT.^1^ Modern techniques such as stereotactic body radiotherapy, image‐guided radiotherapy, and immunotherapy—RT integration have further increased both utilization and complexity. Delays in RT initiation are consistently associated with poorer clinical outcomes, including reduced local control and increased progression, underscoring the need for streamlined care pathways.^2^

Palliative RT accounts for a large proportion of treatments and plays a central role in symptom relief, quality of life improvement, and, in some cases, survival extension.^3^ Spinal metastases are particularly common and clinically urgent, often causing pain, functional decline, or spinal cord compression.^4^ In such cases, timely initiation of spinal RT is critical to prevent neurological deterioration and provide rapid symptom relief.

Current RT workflows require acquisition of a dedicated simulation CT (sCT) for treatment planning. sCTs provide anatomical reference for dose calculation and patient setup but demand an additional in person visit with specialized equipment, flat couch geometry, and immobilization devices.^5^ Compared with widely available diagnostic CT (dCT), sCTs are less accessible and resource‐intensive, and they contribute significantly to delays in treatment initiation.^6^, ^7^, ^8^, ^9^, ^10^, ^11^, ^12^

To address this bottleneck, Diagnostic Scan‐Based Planning (DSBP) approaches have leveraged existing dCT imaging to accelerate planning.^13^, ^14^, ^15^, ^16^, ^17^, ^18^, ^19^ Prospective studies have shown that DSBP can substantially reduce wait times without compromising plan deliverability.^20^, ^21^ However, most of these efforts lacked robust dosimetric comparison between sCT versus dCT, and a fundamental “geometry gap” has not been addressed: Unlike sCT, dCT is typically acquired on curved tabletops without radiotherapy‐specific immobilization, leading to systematic discrepancies in spinal alignment and body contour. Direct use of dCT for dose calculation frequently results in geometric and dosimetric inaccuracies that could exceed clinical tolerances.

The present work departs from conventional synthetic CT (synCT) methodologies. Existing AI‐based synCT studies primarily address intensity‐to‐Hounsfield unit mapping, such as MRI‐only workflows and CBCT correction,^22^, ^23^ these approaches fail to address the underlying physical misalignments inherent in diagnostic positioning.

In this study, we developed a novel AI framework that treats the dCT‐to‐sCT transition not as a pixel‐intensity problem but as a constrained geometric learning task. By modeling systematic, protocol‐dependent offsets in spinal alignment and tabletop curvature, the framework converts dCT into an AI‐adapted planning CT (AI‐pCT) designed to approximate the anatomical and dosimetric characteristics of a dedicated simulation scan.

We evaluated AI‐pCT in a multi‐institutional study of palliative spinal radiotherapy. The model was developed using data from a safety net hospital and externally validated at an independent academic medical center (AMC). Geometric agreement (Dice similarity), dosimetric differences (DVH parameters), and blinded physician assessments were analyzed to test whether AI‐pCT can enable clinically acceptable simulation‐free planning without the loss of treatment accuracy.

## 2 METHOD AND MATERIALS

### 2.1 AI framework

Prior work in geometric learning shows that when two configurations of the same object differ in a structured or rule‐governed manner—due to physical constraints rather than arbitrary variation—the transformation between them can be inferred from data using machine learning models.^24^, ^25^

The similar principle applies to diagnostic versus sCT in spinal radiotherapy. The geometric discrepancy between a diagnostic scan and a simulation scan is not random; it arises systematically from differences in positioning protocol, immobilization, and table geometry. Therefore, the mapping from dCT to a simulation‐equivalent geometry is a constrained and learnable transformation rather than an ill‐posed one.

Formally, the framework models the geometric relationship between diagnostic and simulation configurations as a continuous transformation operator:

(1) $$T_{\theta }\;\left(p\right)=p+\Delta_{\mathrm{sys},\theta }\left(p\right)+\Delta_{\mathrm{pat},\theta }\left(p\right).$$

Where, *p* represents a point in anatomical space, such as a spinal centroid or body‐surface coordinate. And $\Delta_{sys,\theta }$ denotes the deterministic, protocol‐dependent offset caused by systematic differences (e.g., couch curvature and immobilization geometry), while $\Delta_{pat,\theta }$ represents the patient‐specific residual correction learned by the network parameter θ.

The optimization minimizes the mean‐squared deviation between predicted and reference centroid positions:

(2)

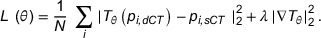

Where the *L*2 regularization term $\lambda |\nabla T_{\theta }{|}_{2}^{2}$ abrupt spatial changes in the learned transformation, promoting anatomically smooth and physically plausible mappings across neighboring tissue centroids.

**FIGURE 1:**
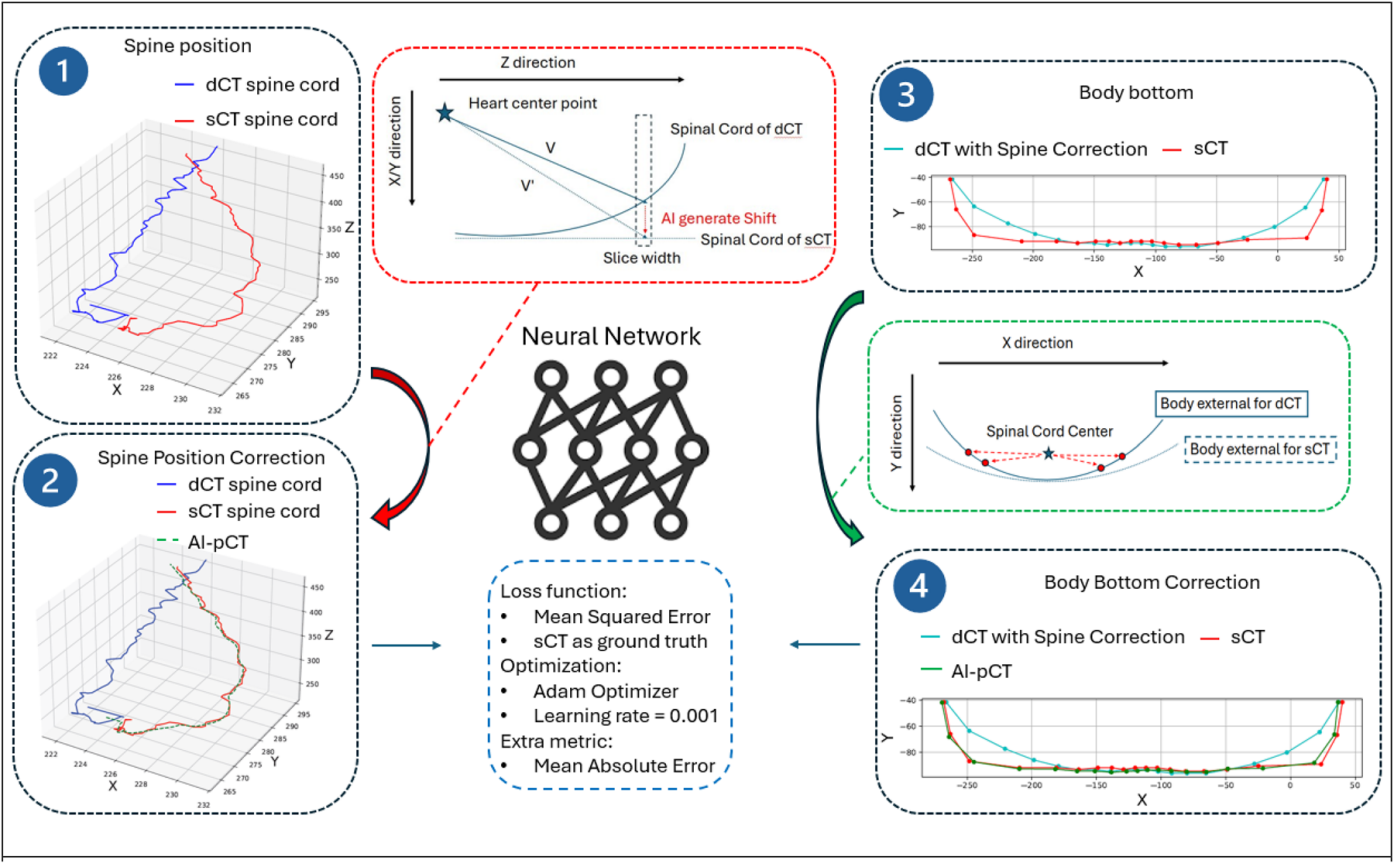
AI workflow for generating AI‐pCT from dCT. The framework learns the geometric transformation from dCT to simulation‐equivalent geometry using paired dCT‐sCT data. (1) The spinal cord trajectory on dCT and sCT is extracted, and the displacement from dCT to sCT is learned using the heart center as an anatomical anchor. (2) The predicted correction is applied to align spine geometry. (3) A second model learns the transformation from curved dCT couch geometry to the flatter sCT body contour using the spinal cord center as reference. (4) The final AI‐pCT combines both corrections. Both networks are trained with sCT as ground truth using MSE loss (Adam optimizer), with MAE recorded as a secondary metric. Coordinate convention: x and y represent the transverse directions on each axial slice, and z denotes the inferior‐superior axis across slices.

Guided by this principle, the proposed AI framework transforms dCT into AI‐pCT by correcting the dominant geometric discrepancies relative to sCT. The model consists of two sequential components: (1) spine position correction and (2) body‐surface correction related to the diagnostic table curvature. A schematic overview of the workflow is shown in Figure 1.

#### 2.1.1 Spine position correction along Z direction

Rigid registration based on bone windows reduces but does not eliminate positional differences between dCT and sCT due to the absence of immobilization devices during diagnostic imaging. To correct these residual misalignments, the algorithm predicts the 3‐D displacement of the spinal cord center between dCT and sCT.

The heart center was defined as the 3D centroid of the physician‐approved heart contour on the dCT. This centroid served as a consistent anatomical reference because the heart maintains a stable position relative to the thoracic vertebrae and can be reliably segmented in both dCT and sCT datasets.

For each axial slice, two vectors are then constructed relative to this heart‐center point:

- $\vec{V}$: from the heart center to the spinal cord center on dCT, and
- $\vec{V^{\prime }}$: from the heart center to the spinal cord center on sCT.
- The displacement vector was defined as $\Delta \;\vec{V}=\vec{V^{\prime }}\;-\vec{V}$

The neural network was trained using:

- Input = [$X_{\mathrm{heart}},Y_{\mathrm{heart}},Z_{\mathrm{heart}}$], $\vec{V}$
- Output = $\Delta \vec{V}$ for that slice

The difference ($\Delta \;\vec{V}=\vec{V^{\prime }}\;-\vec{V}$) represents the displacement required to map the spinal position from dCT to sCT. A neural network is trained to predict ΔV from the heart‐center coordinates and the vector V. The network architecture includes three hidden layers (64‐128‐64 neurons) with ReLU activation, optimized using the Adam optimizer^26^ (learning rate = 0.001, weight decay = 1 × 10^−5^). Weight decay regularization was applied during training to discourage large parameter magnitudes and promote a smooth, generalizable mapping between anatomical coordinates and predicted displacements.

Training is performed patient‐by‐patient rather than slice‐by‐slice to preserve anatomic continuity and capture subject‐specific geometric relationships along the spinal axis. Each patient constitutes one training batch, ensuring that intra‐patient correlations dominate the learned representation rather than random slice‐level noise. The mean‐squared error (MSE) between predicted and reference displacements serves as the loss function, and the mean‐absolute error (MAE) is reported as an auxiliary evaluation metric. The resulting AI‐pCT aligns the spinal axis to the sCT reference geometry (Figure 1, panels 1–2).

#### 2.1.2 Body bottom (table curvature) correction in the *X*‐*Y* plan

dCT scans are typically acquired on a curved tabletop, while sCTs are obtained on a flat treatment couch. This geometric mismatch affects the body's inferior contour and can introduce dose calculation errors. Following spine alignment, the framework applies to a secondary neural network to adjust the body bottom contour of the dCT.

For the second stage, the AI‐corrected spinal cord center serves as the local origin for each axial slice. Body‐surface points from dCT and sCT are represented as vectors [$x_{dCT}\left(\theta \right),y_{dCT}\left(\theta \right)$] and [$x_{sCT}\left(\theta \right),y_{sCT}\left(\theta \right)$], defined as the offsets from the spine center to the external body contour at angle θ. These vectors capture only the geometric difference in table curvature rather than absolute positional differences.

The network was trained slice‐wise to learn the mapping from the dCT contour to the corresponding sCT contour:

Input = [$X_{heart},Y_{heart},Z_{heart}$], $V^{\to }+\Delta V^{\to }$, [$x_{dCT}\left(\theta \right),y_{dCT}\left(\theta \right)$]

Output = [$x_{sCT}\left(\theta \right),y_{sCT}\left(\theta \right)$], for that slice and the same angles θ

For all sampled angles θ∈ [0^°^, 180°]

Here $[X_{heart},Y_{heart},Z_{heart}$] denotes the dCT heart‐center coordinates, which remain constant for each patient across slices; $\left(\vec{V}+\Delta \vec{V}\right)$ is the AI‐corrected spine vector from the heart reference. As with the spine‐correction network, the architecture used three fully connected layers (64‐128‐64 neurons, ReLU activation) and was trained patient‐by‐patient with Adam (learning rate 0.001, weight decay = 1 × 10^−5^) using MSE loss.

The two corrections were applied sequentially: the spine‐correction displacement field was first applied to the dCT, and the body‐surface correction was then applied to the spine‐corrected volume to produce the final AI‐pCT. Formally, if $T_{spine}$ and $T_{body}$ denote the spine correction and body bottom correction transformations, respectively. Then the final AI‐pCT is:

(3) $$\text{AI-pCT}=T_{body}\;\left(T_{spine}\left(\mathrm{dCT}\right)\right)$$

i.e., spine correction is applied first, followed by body‐bottom correction.

### 2.2 Patient cohorts and selection

This retrospective study included 60 patients with spinal metastases treated with palliative radiotherapy across two institutions: a safety net hospital (*n* = 50) and an AMC (*n* = 7). All patients were treated using 3D conformal radiotherapy (3D‐CRT) techniques.

At the safety net hospital, 50 patients were divided into a training/validation cohort (*n* = 42) and an internal test cohort (*n* = 8). Seven AMC patients served as an external test cohort for independent evaluation.

Patient inclusion criteria were as follows:

1. Training/validation cohort (42 safety‐net): dCT scans were required to fully encompass the planning target volume (PTV) and heart.
2. Internal test cohort (*n* = 8) and external test cohort (*n* = 7): in addition to complete PTV and heart coverage, the interval between dCT and sCT had to be less than two weeks to minimize changes in body habitus. This restriction addressed the high risk of weight loss and soft tissue variation in palliative spine patients, which could otherwise compromise geometric and dosimetric comparisons.

For each patient, a dCT and sCT with an existing treatment plan were paired and aligned using frame of reference automated rigid registration based on a bone window kernel in RayStation 11B (RaySearch Laboratories, Stockholm, Sweden). sCTs included pre‐existing manual segmentations performed by the treating radiation oncologist. The heart and spinal cord segmentations on dCTs were segmented automatically using MVision AI Contour+ software (MVision AI, Helsinki, Finland).

The clinically delivered treatment plan parameters were transferred to the dCT and AI‐pCT datasets using the same rigid registration described above. Each treatment plan was recalculated on the dCT, AI‐pCT, and reference sCT using identical clinical dose calculation settings, including heterogeneity corrections and the same beam model, to ensure a fair and consistent dosimetric comparison across image sets. Recalculations were performed in RayStation 11B (RaySearch Laboratories, Stockholm, Sweden) for the safety net cohort and in Eclipse v16.1 (Varian Medical Systems, Palo Alto, CA) for the AMC cohort, maintaining identical beam parameters, monitor units, and dose grid settings to those of the clinically delivered sCT‐based plans.

### 2.3 Evaluation metrics

Dosimetric parameters were extracted to compare the plans, including Dmax, D95, D99, V100, and V107. The root‐mean‐square (RMS) difference in each parameter was calculated to assess variations in the dose‐volume histogram (DVH) curves of the PTV across the plans.

In parallel, plan‐level clinical acceptability was assessed by determining whether all institution‐defined clinical constraints were simultaneously satisfied when the same plan was recalculated on dCT or AI‐pCT, using sCT as the gold standard reference. This direction was chosen for consistency with clinical practice, although the evaluation is mathematically symmetric with respect to dose recalculation across geometries. For goal‐pass analysis, constraints were considered satisfied if within ± 2% of the institutional limit, reflecting the clinical tolerance applied during physician plan approval. Plan‐level assessment was performed only for the Safety Net test cohort, since AMC Eclipse plans did not store clinical goal objects or standardized dose‐constraint definitions applicable across all cases.

Four physicians—four experienced radiation oncologists— blindly evaluated dCT and AI‐pCT plans on a scale of 1 to 5 (5: Perfect, 4: Good, 3: Acceptable, 2: Unacceptable, 1: Poor) based on target and tissue positioning, dose distribution, and clinical goals satisfaction, comparing the plans to the sCT plans as the golden standard.

### 2.4 Statistical analysis

For each patient, paired dosimetric errors were obtained from plans generated on dCT and AI‐pCT using the same sCT‐based plan as reference. Because the paired error distributions were visibly non‐normal, method performance was compared using the Wilcoxon signed‐rank test, the nonparametric analogue of the paired *t*‐test. Statistical significance was defined as *p* < 0.05.

### 2.5 Observer‑agreement analysis

Interobserver variability was assessed using two approaches across all six rater‐pairs.

#### 2.5.1 Simple agreement

- Exact match (%)—for each of the six rater–pair combinations ($C_{4,2}$) the proportion of targets with identical scores was calculated and then averaged over the six pairs.
- ≤ 1‐step agreement (%)—the same procedure, but a pair was counted as agreeing when their scores differed by at most 1 point (e.g. 4 vs 5).

#### 2.5.2 Chance corrected agreement: Gwet's AC^2^ (quadratic weights)

Blinded physician ratings of plan quality were summarized using Gwet's AC^2^, a chance‐corrected agreement coefficient that is more robust than Cohen's κ in the presence of unbalanced categories or high agreement.^27^ We applied quadratic weights for ordinal ratings and interpreted agreement using conventional thresholds (< 0.20 = slight, 0.21–0.40 = fair, 0.41–0.60 = moderate, 0.61–0.80 = substantial, > 0.80 = almost perfect).

## 3 RESULTS

**FIGURE 2:**
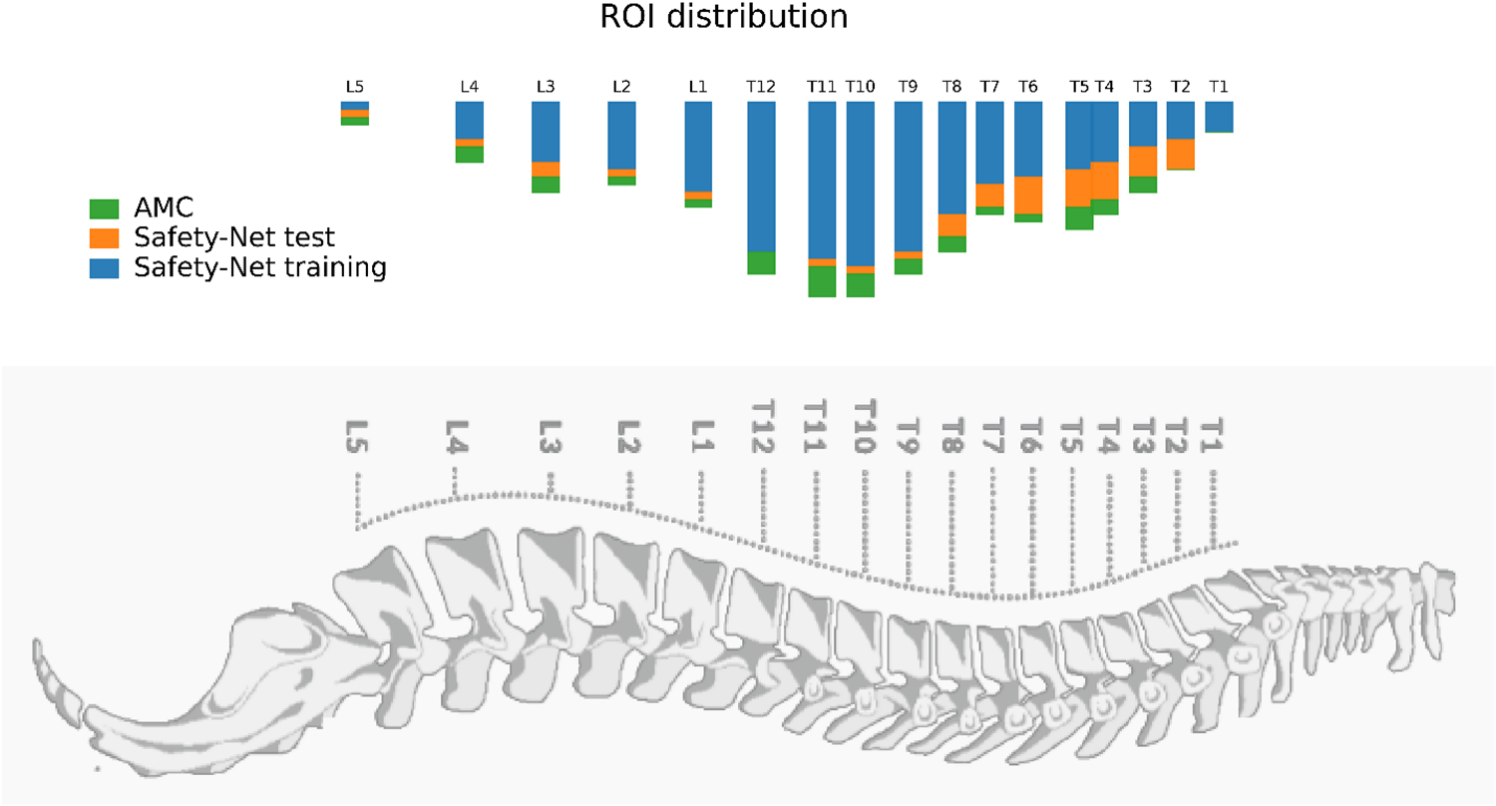
Vertebral‐level distribution of spine ROIs across the safety net training cohort, safety net test cohort, and AMC cohort.

Figure 2 shows the vertebral‐level distribution of ROIs. Most safety net cases involved thoracic targets, with the greatest representation from T4–T12, while lumbar levels were less frequent. Cervical levels were absent in all cohorts because cervical diagnostic scans typically do not include the full heart, which was required for AI input features. The AMC cohort exhibited a similar anatomical distribution, indicating that the model was evaluated on a target pattern comparable to that of the training data.

Tables 1 and 2 summarize the baseline patient characteristics. Median age was similar across cohorts (mid‐50s to early‐60s), while BMI differed substantially between institutions, with the AMC cohort presenting a higher mean BMI than the safety net cohort. The safety net cohort was predominantly male, whereas the AMC cohort demonstrated a more balanced sex distribution. Primary disease sites were dominated by genitourinary, hematologic, and breast cancers in the safety net cohort, whereas the AMC cohort consisted mainly of breast, genitourinary, and gastrointestinal primaries. The overall distribution of disease sites indicates comparable clinical diversity across institutions.

**TABLE 1:** Patient age/BMI from each institution.

Variable	Safety net train	Safety net test	AMC
Age(median [IQR])	56 [47–59]	60 [57–62]	54 [50–62]
BMI (mean ± SD)	24.9 ± 5.2	24.8 ± 5.7	32.4 ± 9.2
Sex	31 Male 11 Female	6 Male 2 Female	3 Male 4 Female

**TABLE 2:** Primary cancer sites of patients from each institution.

	Safety net train	Safety net test	AMC
Genitourinary (GU)	13 (30%)	4 (50%)	1 (14%)
Hematologic	11 (25%)	0	0
Breast	7 (16%)	0	3 (43%)
Thoracic (Lung)	5 (11%)	1 (12%)	1 (14%)
GI	3 (7%)	1 (12%)	1 (14%)
Head & Neck	1 (2%)	1 (12%)	1 (14%)
Gynecologic (GYN)	1 (2%)	0	0
Other	3 (7%)	1 (12%)	0

**FIGURE 3:**
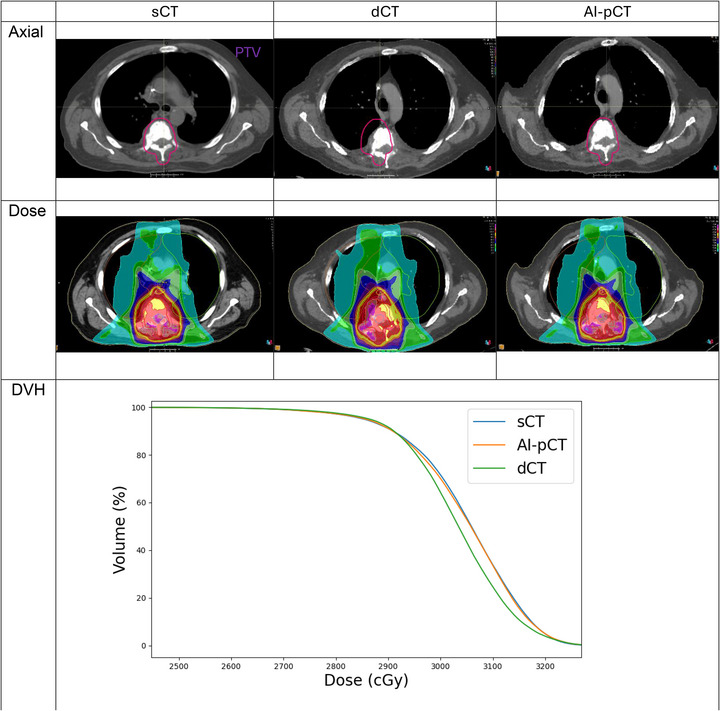
Representative patient example comparing sCT, dCT, and AI‐pCT. Top row: axial images at a matched slice, showing spine misalignment on dCT that is corrected on AI‐pCT. Middle row: corresponding dose distributions using the same treatment plan, illustrating that geometric error on dCT propagates into dose placement and is mitigated after AI correction. Bottom row: Representative DVH for a spinal palliative plan recalculated on sCT, dCT, and AI‐pCT. The dCT‐based plan exhibits inferior target coverage compared with the sCT reference. In contrast, AI‐pCT reduces this discrepancy and more closely reproduces the sCT dose distribution under identical beam parameters.

Figure 3 illustrates a representative case comparing sCT, dCT, and AI‐pCT. In the axial view, the uncorrected dCT shows a visible anterior‐posterior shift of the spine relative to the sCT reference, whereas AI‐pCT restores geometric alignment to match the sCT. The corresponding dose distributions (middle row) demonstrate that this geometric discrepancy in dCT leads to a noticeable shift in isodose coverage, which is largely corrected on AI‐pCT when using the same treatment plan. The accompanying DVH (bottom row) confirms this dosimetric effect quantitatively: dCT deviates from the sCT reference, whereas AI‐pCT closely reproduces the sCT‐based DVH under identical beam parameters.

Table 3 summarizes the structure‐wise geometric accuracy of dCT and AI‐pCT relative to sCT across two independent cohorts (Safety Net and AMC). For each osseous and soft tissue structure, the Dice similarity coefficient and 95th‐percentile Hausdorff distance (HD95) are reported as mean values with 95% confidence intervals.

**TABLE 3:** Structure‐wise *geometric comparison of tissue anatomy between dCT and AI‐pCT, using sCT as reference across two independent cohorts (Safety Net and AMC). The Dice similarity coefficient and 95th‐percentile Hausdorff distance are reported as mean values with 95% CI*.

Safety‐Net	Heart	Kidney left	Kidney right	Liver	Lung left	Lung right	Spinal canal
dCT Dice	0.72 (0.65–0.78)	0.58 (0.44–0.7)	0.52 (0.38–0.65)	0.67 (0.6–0.73)	0.69 (0.56–0.79)	0.65 (0.48–0.8)	0.6 (0.52–0.67)
AI‐pCT Dice	0.72 (0.65–0.78)	0.61 (0.47–0.71)	0.57 (0.43–0.7)	0.67 (0.6–0.75)	0.69 (0.56–0.79)	0.65 (0.48–0.8)	0.88 (0.87–0.9)
dCT HD95 (mm)	12.6 (8.8–17.4)	12.5 (8.4–17)	16.5 (10.5–23)	18.6 (13.7–23.6)	16.9 (9.6–25)	20 (10.1–31.7)	6.9 (5.1–9)
AI‐pCT HD95 (mm)	12.5 (8.6–17.4)	11.5 (7.5–16.3)	15 (8.6–22)	18.5 (13.2–24)	17.1 (9.9–25)	20.2 (10.4–32.2)	1.5 (1.35–1.67)

AMC	Heart	Kidney left	Kidney right	Liver	Lung left	Lung right	Spinal canal
dCT Dice	0.76 (0.66–0.84)	0.56 (0.37–0.7)	0.46 (0.21–0.67)	0.71 (0.62–0.79)	0.66 (0.51–0.82)	0.64 (0.47–0.8)	0.66 (0.53–0.79)
AI‐pCT Dice	0.77 (0.68–0.84)	0.58 (0.37–0.72)	0.49 (0.23–0.71)	0.72 (0.63–0.79)	0.66 (0.5–0.82)	0.64 (0.47–0.8)	0.89 (0.88–0.9)
dCT HD95 (mm)	9.95 (5.72–14.8)	10 (7.8–12.8)	11.6 (6.56–16.6)	17.3 (11.9–23.4)	18.1 (7.3–29)	19.6 (10.1–29.5)	4.4 (2.56–6.5)
AI‐pCT HD95 (mm)	9.15 (5.56–13.3)	9.8 (7.3–13)	11 (6.1–15.9)	17.4 (11.7–24)	18 (7.4–29)	20.2 (10.4–30.4)	1.22 (1.2–1.26)

AI‐pCT demonstrates substantial geometric improvement for the spinal canal (dice from 0.6 to 0.88, HD95: 7 mm to 1.5 mm), whereas geometric metrics for other soft tissue organs (heart, kidneys, liver, lungs) show minimal change relative to dCT. Meanwhile, the magnitude and pattern of these structure‐specific effects are highly consistent between cohorts, indicating robust and reproducible geometric behavior across institutions.

**FIGURE 4:**
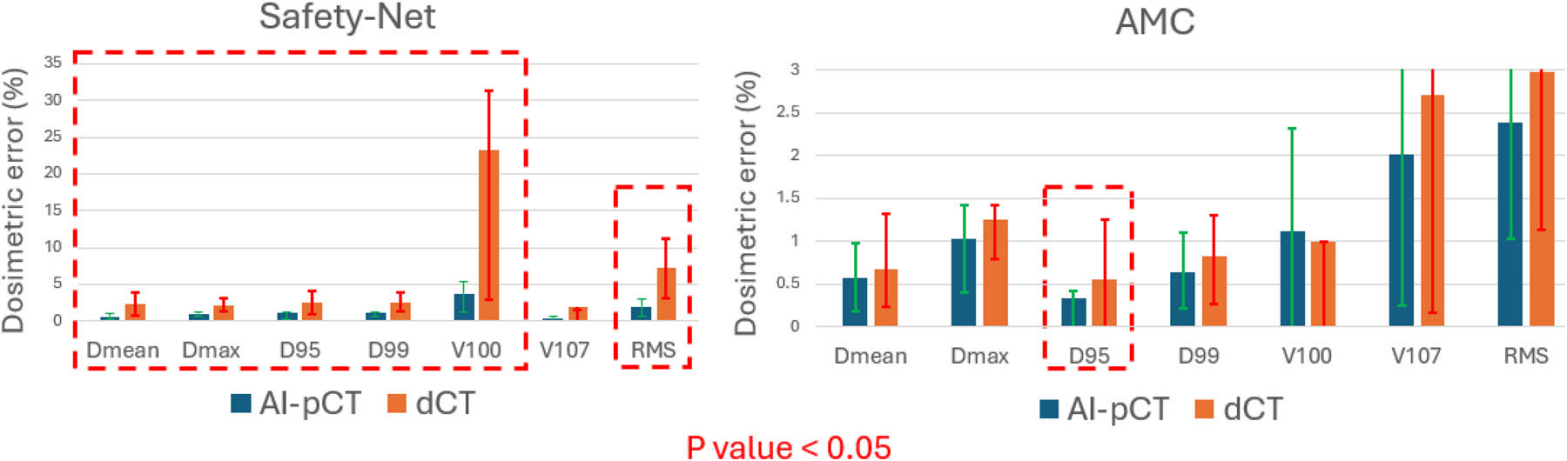
Mean differences in DVH metrics (vs. sCT) for plans recalculated on dCT and AI‐pCT in the safety net cohort (left) and AMC cohort (right). Red dashed boxes highlight metrics with significant improvement (*p* < 0.05).

Figure 4 compares the dosimetric deviations from sCT when plans are recalculated on dCT versus AI‐pCT. Using the same treatment plan as reference, dCT exhibited substantially larger errors—most notably in V100 and RMS—whereas AI‐pCT consistently reduced these deviations across both institutions. The largest impact was observed in the safety net cohort, where uncorrected dCT misalignment was greatest. Differences were statistically significant for most metrics (Wilcoxon signed‐rank, *p* < 0.05). Consistent with these improvements, and applying a ± 2% clinical tolerance consistent with physician acceptance criteria, as shown in Table 4, plan‐level goal achievement in the safety net test cohort increased from 37.5% with dCT to 100% with AI‐pCT (Table 4).

**TABLE 4:** Plan‐level clinical goal achievement (Safety Net test cohort, *n* = 8). Plans meeting all institutional constraints (± 2% tolerance) increased from 37.5% with dCT to 100% with AI‐pCT.

Safety Net test *n* = 8	dCT	AI‐pCT
Plan‐level goal pass rate	37.5% (3/8)	100% (8/8)

**FIGURE 5:**
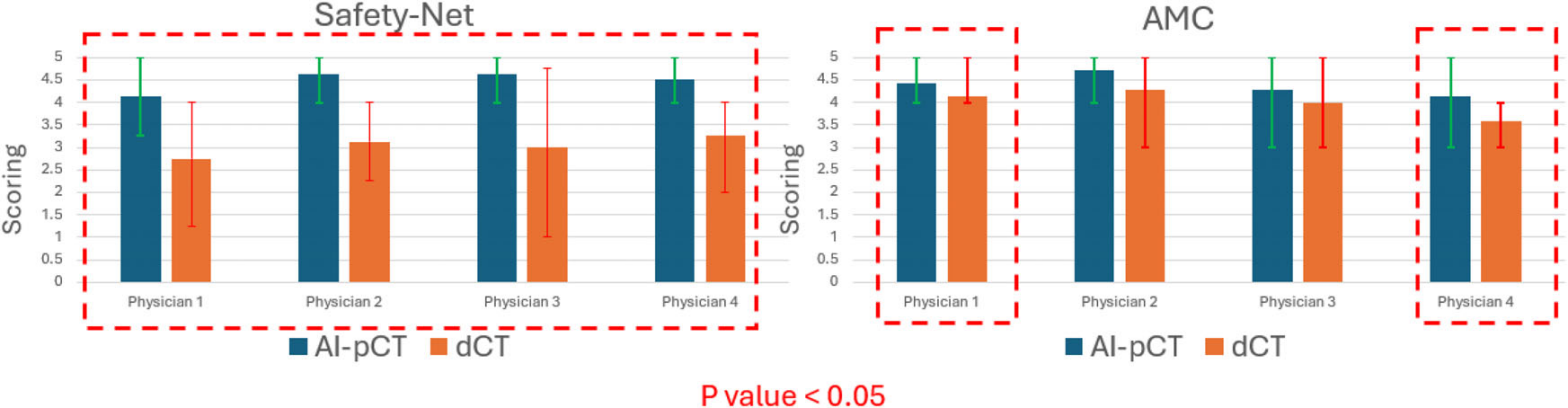
Physician scoring of dCT versus AI‐pCT for treatment planning across safety net (left) and AMC (right) cohorts. Red dashed boxes highlight metrics with significant improvement (*p* < 0.05).

Figure 5 shows physician scoring of image suitability for treatment planning on dCT versus AI‐pCT for both cohorts. Across all four physicians and both institutions, AI‐pCT received consistently higher ratings, with significantly higher scores observed in the safety net cohort (*p* < 0.05). Error bars denote interobserver variability. Table 5 summarizes interobserver agreement, showing that AI‐pCT yielded a higher exact‐match rate (56% vs. 38%) and a higher near‐agreement rate (≤1‐step difference: 98% vs. 87%). Gwet's AC^2^ indicated moderate chance‐corrected agreement for both image types.

**TABLE 5:** Interobserver agreement for four physicians evaluating 15 cases (from two institutions) on dCT and AI‐pCT.

Interobserver agreement	% exact match	% ≤ 1‐step	Gwet's AC^2^ (quadratic)
dCT	38	87	0.53
AI‐pCT	56	98	0.53

## 4 DISCUSSION

The geometric discrepancy between dCT and sCT arises primarily from differences in acquisition protocols and immobilization: dCT is obtained on a curved diagnostic couch without treatment positioning devices, whereas sCT is acquired on a flat couch with immobilization (e.g., masks or body molds) to replicate treatment conditions. As shown in the left panels of Figure 1, the resulting spinal misalignment cannot be resolved by rigid transformation alone. In practical terms, this means that the same discrepancy cannot be corrected at the treatment machine using couch shifts or rotations.

This limitation is particularly relevant in the palliative spine setting, where metastatic disease frequently spans multiple contiguous vertebral levels or involves multiple sites along the spinal axis, making extended longitudinal targets common in routine clinical practice. Because palliative spine radiotherapy commonly involves extended or multisite targets, geometric accuracy outside a single anchor level is clinically relevant rather than incidental. For targets spanning multiple vertebral levels, such residual nonrigid error would require repeated couch repositioning along the spinal axis if dCT were used for planning—a clinically infeasible workflow. As an illustrative example from the safety net test cohort, constraining rigid registration to the target region reduced local alignment error at the anchor level but increased curvature‐induced residual displacement at non‐anchored levels, with the maximum intervertebral deviation of the spinal cord center increasing from a median of 6.7 mm [IQR: 4.15–11.05] to 8.23 mm [IQR: 5.20–11.50]. This behavior reflects the intrinsic limitation of rigid registration for extended spinal targets and directly motivates the use of AI‐pCT to achieve anatomically consistent, global geometric correction rather than relying on local rigid alignment alone.

The success of the AI models in correcting both spine position and body contour demonstrates that a learnable, deterministic correlation exists between dCT and sCT geometry. This is expected, as both dCT acquisition and RT simulation follow standardized protocols within the same institution. In effect, the model functions as a mathematical surrogate for the physical constraints that govern the relationship between dCT and sCT—including the predictable effect of gravity on body contour and the systematic difference between curved diagnostic tables and flat treatment couches.

The impact of spine position and table curvature adjustments is evident in the patient case presented in Figure 3. AI‐pCT demonstrates strong alignment in spinal cord position and GTV with sCT under the treatment setup, whereas dCT exhibits a noticeable deviation in spinal cord position relative to sCT. The dose distribution difference between dCT and sCT is less pronounced than the spatial discrepancy. However, the dose distribution on dCT is colder on the left side of the GTV compared to sCT, indicating that using dCT for treatment planning could result in an overdosed target during delivery.

To generalize these observations beyond a single case, we performed a structure‐wise geometric analysis (Table 3) to elucidate *why* and *under what conditions* the proposed AI‐pCT approach is effective. Substantial geometric improvement was observed for the spinal canal, an anatomy that is strongly coupled with vertebral curvature and therefore particularly sensitive to differences in patient positioning and couch geometry between diagnostic and sCT. In contrast, geometric metrics for other soft tissue organs (e.g., heart, kidneys, liver, and lungs) showed minimal change relative to dCT, indicating that residual misalignment of these organs with respect to sCT is not primarily driven by vertebral curvature or couch‐induced deformation.

In spinal palliative radiotherapy, such residual soft tissue discrepancies are generally clinically acceptable, as treatment targets are dominated by osseous anatomy and spinal canal constraints. However, these findings also highlight an important limitation: correcting vertebral curvature and couch geometry alone may be insufficient for applications requiring higher geometric precision, such as stereotactic body radiation therapy (SBRT), where soft tissue alignment plays a more critical role.

Notably, the magnitude and pattern of these structure‐wise geometric metrics were highly consistent across two independent cohorts, despite differences in patient demographics, imaging protocols, and institutional practice. This consistency suggests that while soft tissue positional differences between diagnostic and sCT may vary on a per‐patient basis, their overall deviation range is reproducible and systematic at the population level. Future work may therefore focus on integrating AI‐pCT‐based geometric correction with probabilistic models of organ motion or uncertainty‐aware planning strategies to support more precision‐sensitive treatment paradigms.

Consistent with these geometric observations, the dosimetric impact of AI‐pCT is most pronounced in settings where dCT‐sCT discrepancies are large. As shown in Figure 4, using sCT as the reference and keeping the treatment plan fixed, AI‐pCT consistently reduced dosimetric error in the safety net cohort across all endpoints, with narrower inter‐patient variability. For example, the mean dose error (Dmean) decreased from 2.4% on dCT to 0.57% on AI‐pCT (*p* = 0.004), and the global DVH deviation (RMS) was reduced from 7.3% to 2.0% (*p* = 0.004). The largest change was observed in V100, which dropped from 23.2% to 3.7% (*p* = 0.014). These findings confirm that correcting spinal position and table‐curvature effects materially improves dosimetric fidelity when dCT‐sCT deviations are substantial.

A key observation is that the dosimetric improvements with AI‐pCT were not merely numerical; they changed clinical decision‐making outcomes. As shown in Table 4, in the safety net cohort, image correction converted many otherwise‐unacceptable dCT plans into clinically acceptable ones without replanning. This indicates that AI‐pCT does not just reduce geometric error—it shifts cases across the clinical acceptance boundary, which is the practical threshold for eliminating a sCT.

In the AMC cohort, the improvement was smaller and often not statistically significant because the baseline dCT geometry was already close to sCT (e.g., mean DVH RMS = 2.97%), which is likely due to the institution's stricter pre‐scan alignment protocol. Thus, AI‐pCT had less opportunity to produce a measurable gain in this setting.

A similar trend was observed in physician scoring (Figure 5). In the safety net cohort, average ratings increased from “Acceptable” (2.7–3.1) on dCT to “Good–Perfect” (4.1–4.7) on AI‐pCT, with statistically significant improvement for all observers. In the AMC cohort, baseline dCT scores were already higher (3.4–4.2), yet AI‐pCT still yielded significant improvement for two observers and maintained at least “Good” quality for the others. Taken together, these data indicate that AI‐pCT provides the greatest absolute benefit in suboptimized acquisition environments, while still offering non‐inferior or modestly improved performance under best‐practice conditions.

**FIGURE 6:**
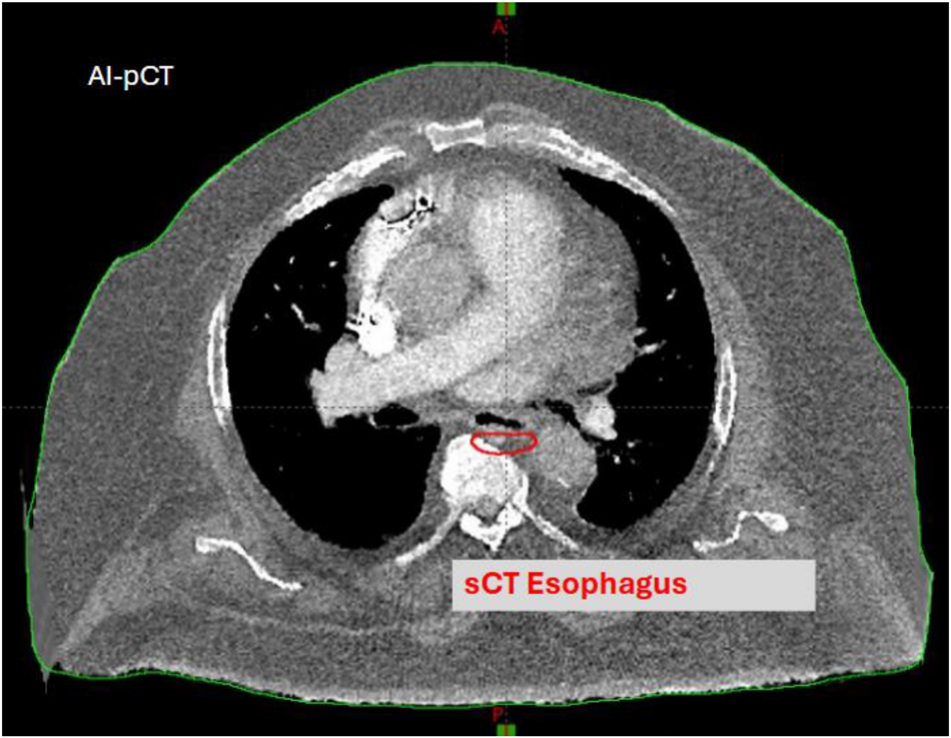
Example of a residual soft tissue misalignment after AI‐pCT correction. The red contour highlights the esophagus on the sCT, showing that small local deviations may persist even when global spine geometry is corrected.

This study has several limitations. Although correction of spine position and table curvature greatly reduced PTV misalignment, mobile soft tissue structures—particularly small, deformable organs such as the esophagus—remain difficult to align, as illustrated in Figure 6. During blinded review, several physicians noted residual offsets between AI‐pCT and sCT contours in these regions, which could become clinically relevant in treatments with tighter margins. Second, all patients in this study were treated using 3D conformal palliative techniques; therefore, the results may not directly extrapolate to high‐precision modalities such as VMAT or SBRT, where submillimeter geometric fidelity is required. Third, the current workflow excludes cervical‐spine cases because the model relies on heart anatomy as an anchoring feature, which is not available in cervical‐only diagnostic scans.

Future work will extend this framework in three directions. First, while perfect prediction of mobile soft tissue position is likely unattainable, generating a probabilistic envelope of plausible locations may still be clinically valuable for margin design and robustness evaluation. Second, validation will be extended to high‐precision techniques such as VMAT and SBRT, where tighter margins and steeper dose gradients provide a stricter test of whether AI‐pCT is sufficient to replace sCT. Third, generalization beyond thoracic and lumbar targets will be pursued by incorporating alternative anatomical anchors for cervical‐spine cases to enable extension across the full vertebral column. In parallel, future clinical deployment could leverage on board CBCT at the first fraction as an independent geometric validation step for AI‐pCT, providing an opportunity to confirm anatomical fidelity before committing to a simulation‐free workflow.

## 5 CONCLUSION

This study demonstrates that AI‐based correction of dCT can recover simulation‐equivalent geometry by jointly adjusting spinal alignment and body contour, overcoming the primary barriers that have historically limited the use of dCT for palliative spine planning. Across cohorts, AI‐pCT reduced the Dmean from 2.4% to 0.57% and the global DVH deviation (RMS) from 7.3% to 2.0% (*p* < 0.01), while improving physician plan‐quality ratings from “Acceptable” (mean = 3.0) to “Good‐Perfect” (mean = 4.4). Plan‐level clinical‐goal achievement increased from 37.5% to 100%, with the largest benefit observed in the safety net cohort, where dCT‐sCT geometric discrepancies were greatest. These results confirm that AI‐pCT achieves sCT‐level geometric and dosimetric fidelity without requiring a separate simulation scan, providing a viable pathway toward simulation‐free planning for spinal palliative radiotherapy. Future work will extend validation to high‐precision modalities (VMAT, SBRT) and incorporate soft tissue modeling to establish a robust, generalizable framework for simulation‐free RT.

## CONFLICT OF INTEREST STATEMENT

No conflict of interest needs to be reported.

## Data Availability

This study was based on the Baylor St Luke's Medical Center and Smith Clinic data. The authors do not own these data and hence are not permitted to share them in the original form (only in aggregate form, e.g., publications). *Research data are stored in an institutional repository and will be shared upon request to the corresponding author*.

## References

[1] Atun R , Jaffray DA , Barton MB , et al. Expanding global access to radiotherapy. Lancet Oncol. 2015;16(10):1153–1186. doi:10.1016/S1470‐2045(15)00222‐3 26419354 10.1016/S1470-2045(15)00222-3

[2] Robinson D , Massey T , Davies E , Jack RH , Sehgal A , Møller H . Waiting times for radiotherapy: variation over time and between cancer networks in southeast England. Br. J. Cancer. 2005;92(7):1201–1208. doi:10.1038/sj.bjc.6602463 15785752 10.1038/sj.bjc.6602463 PMC2361967

[3] International Atomic Energy Agency Radiotherapy in palliative cancer care: development and implementation. 2012;International Atomic Energy Agency.

[4] Davis DD , Kane SM . Palliation radiation therapy of the spinal cord. In StatPearls 2025;StatPearls Publishing LLC. 32644677

[5] Hegi F , Atwood T , Keall P , Loo BW . 34 ‐ Technical requirements for lung cancer radiotherapy. In: Pass H.I. , Ball D. , Scagliotti G.V. , eds. IASLC Thoracic Oncology. 2nd ed. Elsevier; 2018:318–329.e2.

[6] Benk V . Effect of delay in initiating radiotherapy for patients with early stage breast cancer. Clin. Oncol. 2004;16(1):6–11. doi:10.1016/j.clon.2003.10.008 10.1016/j.clon.2003.10.008 14768749

[7] Huang J , Barbera L , Brouwers M , Browman G , Mackillop WJ . Does delay in starting treatment affect the outcomes of radiotherapy? A systematic review. J. Clin. Oncol. 2003;21(3):555–563. doi:10.1200/JCO.2003.04.171 12560449 10.1200/JCO.2003.04.171

[8] Everitt S , Herschtal A , Callahan J , et al. High rates of tumor growth and disease progression detected on serial pretreatment fluorodeoxyglucose‐positron emission tomography/computed tomography scans in radical radiotherapy candidates with nonsmall cell lung cancer. Cancer. 2010;116(21):5030–5037. doi:10.1002/cncr.25392 20623786 10.1002/cncr.25392

[9] Do V , Gebski V , Barton MB . The effect of waiting for radiotherapy for grade III/IV gliomas. Radiother Oncol. 2000;57(2):131–136. doi:10.1016/S0167‐8140(00)00257‐7 11054516 10.1016/s0167-8140(00)00257-7

[10] Buszek SM , Al Feghali KA , Elhalawani H , Chevli N , Allen PK , Chung C . Optimal timing of radiotherapy following gross total or subtotal resection of glioblastoma: a real‐world assessment using the national cancer database. Sci. Rep. 2020;10(1):4926. doi:10.1038/s41598‐020‐61701‐z 32188907 10.1038/s41598-020-61701-z PMC7080722

[11] Jensen AR , Nellemann HM , Overgaard J . Tumor progression in waiting time for radiotherapy in head and neck cancer. Radiother Oncol. 2007;84(1):5–10. doi:10.1016/j.radonc.2007.04.001 17493700 10.1016/j.radonc.2007.04.001

[12] Žumer B , Pohar Perme M , Jereb S , Strojan P . Impact of delays in radiotherapy of head and neck cancer on outcome. Radiat Oncol. 2020;15(1):202. 32819389 10.1186/s13014-020-01645-w PMC7441656

[13] Suzuki K , Hirasawa Y , Yaegashi Y , Miyamoto H , Shirato H . A web‐based remote radiation treatment planning system using the remote desktop function of a computer operating system: a preliminary report. J Telemed Telecare. 2009;15(8):414–418. doi:10.1258/jtt.2009.090409 19948709 10.1258/jtt.2009.090409

[14] Norum J , Bruland ØS , Spanne O , et al. Telemedicine in radiotherapy: a study exploring remote treatment planning, supervision and economics. J Telemed Telecare. 2005;11(5):245–250. doi:10.1258/1357633054471858 16035967 10.1258/1357633054471858

[15] Belard A , Dolney D , Zelig T , McDonough J , O'Connell J . Improving proton therapy accessibility through seamless electronic integration of remote treatment planning sites. Telemed J E Health. 2011;17(5):370–375. doi:10.1089/tmj.2010.0199 21492029 10.1089/tmj.2010.0199

[16] Glober G , Kubli A , Kielbasa J et al. Technical report: diagnostic scan‐based planning (DSBP), a method to improve the speed and safety of radiation therapy for the treatment of critically Ill patients. Pract. Radiat. Oncol. 2020;10(5):e425–e431. doi:10.1016/j.prro.2020.01.009 32004703 10.1016/j.prro.2020.01.009

[17] Wong S , Roderick S , Kejda A et al. Diagnostic computed tomography enabled planning for palliative radiation therapy: removing the need for a planning computed tomography scan. Pract. Radiat. Oncol. 2021;11(2):e146–e153. doi:10.1016/j.prro.2020.10.010 33186781 10.1016/j.prro.2020.10.010

[18] Schiff JP , Zhao T , Huang Yi , Sun B , Hugo GD , Spraker MB , Abraham CD . Simulation‐free radiation therapy: an emerging form of treatment planning to expedite plan generation for patients receiving palliative radiation therapy. Adv. Radiat. Oncol. 2023;8(1):101091. doi:10.1016/j.adro.2022.101091 36304132 10.1016/j.adro.2022.101091 PMC9594122

[19] Nelissen KJ , Versteijne E , Senan S , et al. Same‐day adaptive palliative radiotherapy without prior CT simulation: early outcomes in the FAST‐METS study. Radiother Oncol. 2023;182:109538. doi:10.1016/j.radonc.2023.109538 36806603 10.1016/j.radonc.2023.109538

[20] Schuler T , Roderick S , Wong S , et al., Real‐World implementation of simulation‐free radiation therapy (SFRT‐1000): a propensity score‐matched analysis of 1000 consecutive palliative courses delivered in routine care. Int. J. Radiat. Oncol. Biol. Phys. 2025;121(3):585–595. doi:10.1016/j.ijrobp.2024.09.041 39353478 10.1016/j.ijrobp.2024.09.041

[21] O'Neil M , Laba JM , Nguyen TK , et al. Diagnostic CT‐Enabled planning (DART): Results of a randomized trial in palliative radiation therapy. Int. J. Radiat. Oncol. Biol. Phys. 2024;120(1):69–76. doi:10.1016/j.ijrobp.2024.03.005 38613562 10.1016/j.ijrobp.2024.03.005

[22] Bahloul MA , Jabeen S , Benoumhani S , Alsaleh HA , Belkhatir Z , Al‐Wabil A . Advancements in synthetic CT generation from MRI: a review of techniques, and trends in radiation therapy planning. J. Appl. Clin. Med. Phys. 2024;25(11):e14499. doi:10.1002/acm2.14499 39325781 10.1002/acm2.14499 PMC11539972

[23] Bornstein MM , Horner K , Jacobs R . Use of cone beam computed tomography in implant dentistry: current concepts, indications and limitations for clinical practice and research. Periodontology 2000. 2017;73(1):51–72. doi:10.1111/prd.12161 28000270 10.1111/prd.12161

[24] Bronstein MM , Bruna J , Cohen T , Veličković P . Geometric deep learning: grids, groups, graphs, geodesics, and gauges. arXiv preprint arXiv:2104.13478, 2021.

[25] Borde HSdO , Bronstein M . Mathematical foundations of geometric deep learning. arXiv preprint arXiv:2508.02723, 2025.

[26] Kingma DP . Adam: a method for stochastic optimization. arXiv preprint arXiv:1412.6980, 2014.

[27] Gwet K . Handbook of inter‐rater reliability: *t*he definitive guide to measuring the extent of agreement among raters. 2012; Advanced Analytics, LLC.

